# Some putative prebiotics increase the severity of *Salmonella enterica *serovar Typhimurium infection in mice

**DOI:** 10.1186/1471-2180-9-245

**Published:** 2009-11-30

**Authors:** Anne Petersen, Peter MH Heegaard, Anna L Pedersen, Jens B Andersen, Rikke B Sørensen, Hanne Frøkiær, Sampo J Lahtinen, Arthur C Ouwehand, Morten Poulsen, Tine R Licht

**Affiliations:** 1 The National Food Institute, Department of Microbiology and Risk Assessment, Technical University of Denmark, Moerkhoej Bygade 19, DK-2860 Soeborg, Denmark; 2 The National Veterinary Institute, Department of Veterinary Diagnostics and Research, Technical University of Denmark, Bülowsvej 27, DK-1790 Copenhagen V, Denmark; 3 Department of Systems Biology, Center for Biological Sequence Analysis, Technical University of Denmark, Soeltofts Plads, Building 221, DK-2800 Kgs. Lyngby, Denmark; 4 Department of Basic Sciences and Environment Faculty of Life Sciences Copenhagen University Thorvaldsensvej 40, DK-1871 Frederiksberg C, Denmark; 5 Danisco Health & Nutrition, Sokeritehtaantie 20, 02460 Kantvik, Finland

## Abstract

**Background:**

Prebiotics are non-digestible food ingredients believed to beneficially affect host health by selectively stimulating the growth of the beneficial bacteria residing in the gut. Such beneficial bacteria have been reported to protect against pathogenic infections. However, contradicting results on prevention of *Salmonella *infections with prebiotics have been published. The aim of the present study was to examine whether *S*. Typhimurium SL1344 infection in mice could be prevented by administration of dietary carbohydrates with different structures and digestibility profiles. BALB/c mice were fed a diet containing 10% of either of the following carbohydrates: inulin, fructo-oligosaccharide, xylo-oligosaccharide, galacto-oligosaccharide, apple pectin, polydextrose or beta-glucan for three weeks prior to oral *Salmonella *challenge (10^7 ^CFU) and compared to mice fed a cornstarch-based control diet.

**Results:**

The mice fed with diets containing fructo-oligosaccharide (FOS) or xylo-oligosaccharide (XOS) had significantly higher (P < 0.01 and P < 0.05) numbers of *S*. Typhimurium SL1344 in liver, spleen and mesenteric lymph nodes when compared to the mice fed with the cornstarch-based control diet. Significantly increased amounts (P < 0.01) of *Salmonella *were detected in ileal and fecal contents of mice fed with diets supplemented with apple pectin, however these mice did not show significantly higher numbers of S. Typhimyrium in liver, spleen and lymph nodes than animals from the control group (P < 0.20).

The acute-phase protein haptoglobin was a good marker for translocation of *S*. Typhimurium in mice. In accordance with the increased counts of *Salmonella *in the organs, serum concentrations of haptoglobin were significantly increased in the mice fed with FOS or XOS (P < 0.001). Caecum weight was increased in the mice fed with FOS (P < 0.01), XOS (P < 0.01), or polydextrose (P < 0.001), and caecal pH was reduced in the mice fed with polydextrose (P < 0.001). *In vitro *fermentation in monocultures revealed that *S*. Typhimurium SL1344 is capable of fermenting FOS, beta-glucan and GOS with a corresponding decline in pH.

**Conclusion:**

Supplementing a cornstarch-based rodent diet with 10% FOS or XOS was found to increase the translocation of *S*. Typhimurium SL1344 to internal organs in mice, while 10% apple pectin was found to increase the numbers of S. Typhimurium in intestinal content and feces.

## Background

One of the basic physiological functions of the resident microbiota is that it functions as a microbial barrier against pathogens [1]. A healthy, balanced microbiota has been suggested to be predominantly saccharolytic, with significant numbers of bifidobacteria and lactobacilli [2]. The use of pre- and probiotics has thus been suggested as approaches to prevent *Salmonella *infections and infections by enteric pathogens in general [3-5].

Prebiotics were originally defined as "non-digestible food ingredients that beneficially affect the host by selectively stimulating the growth and/or activity of one or a limited number of bacteria in the colon, and thus improve host health" [6]. The main candidates that meet the required criteria for classification of a food ingredient as a prebiotic are fructo-oligosaccharides, including inulin, galacto-oligosaccharides and lactulose [7]. Numerous studies have shown that prebiotics stimulate the growth of bifidobacteria and lactobacilli *in vivo *[8-12] and specific strains from these genera have been shown to suppress bacterial infections including those caused by ingestion of *Salmonella enterica *serovar Typhimurium (*S*. Typhimurium) [13-17]. Mechanisms proposed to explain the enhanced resistance to pathogens induced by lactobacilli and bifidobacteria include (i) competitive inhibition of the epithelial and mucosal adherence of pathogens, (ii) production of antimicrobial substances, (iii) immune modulation, and (iv) production of short chain fatty acids which can reduce the growth of acid-sensitive pathogens like *Salmonella *[1,18,19].

*Salmonella *infections are a global problem with *Salmonella enterica *serovar Typhi (*S*. Typhi) and serovar Paratyphi (*S*. Paratyphi) causing epidemics of severe systemic infections in developing countries [20,21]. *S*. Typhi and *S*. Paratyphi do not cause systemic infections in other mammalian hosts than humans, but the BALB/c mouse model used in the present study provides a murine model of human typhoid fever [22]. In the EU, *Salmonella enterica *serovar Enteritidis (*S*. Enteritidis) and *S*. Typhimurium are the most frequently reported serovars causing human salmonellosis. A total number of 160.649 cases of human salmonellosis were reported in the EU in 2006 [23].

Despite the promising effects of probiotics on the prevention of *Salmonella *infections in mice [13,14,17,24], studies with prebiotics have shown conflicting results. Inulin has been found to reduce the mortality of mice challenged with *S*. Typhimurium [25] and in rats fed an inulin-oligofructose diet, numbers of *S*. Typhimurium in the content of ileum and caecum were reduced [26]. Additionally, increased resistance to *S*. Typhimurium infection in mice was reported with combined administration of bifidobacteria and galacto-oligosaccharides [15]. Finally, a recent study showed that oral administration of galacto-oligosaccharides to mice immediately prior to *S*. Typhimurium SL1344 infection reduced the clinical signs of infection, significantly reduced the organ counts of *S*. Typhimurium, and reduced the pathology associated with murine salmonellosis [27]. In contrast to these findings, a number of papers reporting an increased translocation of *S*. Enteritidis in rats fed inulin, fructo-oligosaccharides or lactulose have been published by one group of investigators [28-31]. However, these studies were all based on low calcium-diets and the adverse effect could be reversed by oral administration of calcium [31].

The aim of the present study was to examine if mouse susceptibility to *S*. Typhimurium SL1344 infection was affected by ingestion of carbohydrates with different structures and digestibility profiles. Effects of diets containing inulin, fructo-oligosaccharide (FOS), xylo-oligosaccharide (XOS), galacto-oligosaccharide (GOS), apple pectin, polydextrose or beta-glucan on murine *S*. Typhimurium infection were compared to a cornstarch-based control diet. This is, to our knowledge, the first study comparing the effects of non-digestible carbohydrates with different structures on *Salmonella *infection.

## Results

### Body weight and euthanisation

To monitor the effect of feeding with different potentially prebiotic carbohydrates on the susceptibility to infection with S. Typhimurium, groups of mice were fed with diets containing either of the seven abovementioned carbohydrates for three weeks prior to challenge with *Salmonella*.

During the three weeks of feeding on the experimental diets, no significant differences in mean body weights were recorded between the dietary groups. Following the *Salmonella *challenge, the mice were monitored and euthanized before schedule in case of adverse signs of infection due to ethical considerations.

Only mice euthanised as scheduled on Day 5 were included in the analysis. These constituted five mice in the group fed polydextrose, six mice in the groups fed apple pectin, beta-glucan and GOS, seven mice in the groups fed XOS and control diet (study B), and all mice in the remaining groups (inulin, FOS and control diet in study A+C).

### Caecum weight and pH

The weight of caecum was significantly increased in mice fed diets containing FOS (P < 0.01), XOS (P < 0.01) or polydextrose (P < 0.001) when compared to groups fed the control diet (Table 1). Polydextrose ingestion was found to decrease (P < 0.001) the caecal pH (Table 1).

**Table 1:** Weight and pH of caecum five days post challenge^a^

	N^b^	Caecum weight incl. content (mg)	pH of caecal content
			

**Study A:**			

**Control**	7	198.96 ± 14.15	7.52 ± 0.06

**FOS**	10	355.32 ± 32.09**	7.72 ± 0.19

**XOS**	7	358.74 ± 44.66**	7.45 ± 0.25

			

**Study B:**			

**Control**	7	181.70 ± 10.60	7.08 ± 0.12

**Beta-glucan**	6	206.40 ± 76.03	6.85 ± 0.17

**GOS**	6	174.83 ± 38.95	7.07 ± 0.15

			

**Study C:**			

**Control**	8	205.36 ± 20.93	7.17 ± 0.05

**Inulin**	8	263.24 ± 24.05	7.07 ± 0.09

**Apple pectin**	6	216.68 ± 18.20	7.02 ± 0.14

**Polydextrose**	5	637.74 ± 61.11***	6.60 ± 0.05***

^a^Values represent means ± SEM. ^b^Group size on Day 5 post challenge. One mouse died during the acclimatisation period in the control group in study A. **P < 0.01; ***P < 0.001.

### *Salmonella *cultivated from faecal samples and distal part of ileum

**Figure 1:**
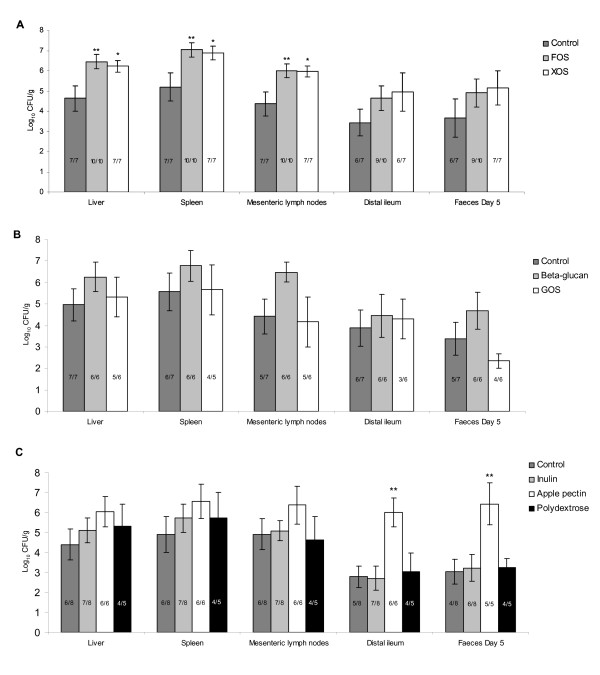
***Salmonella *counts in organs, distal ileum, and faeces**. Enumeration of *S*. Typhimurium SL1344 from the liver, spleen, mesenteric lymph nodes, distal part of ileum and faeces from mice five days post challenge. A: Control, FOS and XOS; B: Control, beta-glucan and GOS; C: Control, inulin, apple pectin and polydextrose. Values represent means ± SEM. Prevalences of mice with detectable numbers of *Salmonella *in the organs are shown on the columns. *P < 0.05; **P < 0.01

There was a trend (Figure 1), though not statistically significant, indicating that faecal counts of *S*. Typhimurium cultivated from faecal samples were higher on Day 3 after challenge in the groups fed FOS (P = 0.068) and XOS (P = 0.066) when compared to the group fed the control diet. (Data not shown). In mice fed apple pectin, faecal counts of *S*. Typhimurium were significantly higher on Day 3 (P < 0.01) and Day 5 (P < 0.01) (Figure 1C). The increased faecal counts in the apple pectin group corresponded to a significantly higher number of *S*. Typhimurium in the content of the distal part of ileum at euthanisation on Day 5 (P < 0.01). Also in the FOS and XOS group, there was a trend that ileal *Salmonella *counts were elevated (P = 0.182 and P = 0.242, respectively), though this was not statistically significant (Figure 1A).

Feeding with beta-glucan and GOS did not significantly affect the ileal and fecal numbers of *Salmonella *when compared to the control (Figure 1B).

### *Salmonella *cultivated from liver, spleen and mesenteric lymph nodes

Numbers of *S*. Typhimurium cultivated from the liver, spleen and mesenteric lymph nodes were significantly higher in mice fed FOS (P < 0.01) or XOS (P < 0.05) with an increase in the mean CFU counts of approximately 1.6 to1.8 logs (Figure 1A). In animals fed with apple pectin, a similar trend showing increased counts of *Salmonella *in liver (P = 0.154) and spleen (P = 0.198) was observed.

Feeding with beta-glucan and GOS did not significantly affect the numbers of *Salmonella *in the investigated organs when compared to the control (Figure 1B).

### Serum levels of haptoglobin

In all dietary groups the concentration of serum haptoglobin was markedly and significantly elevated by *Salmonella *challenge (Table 2). The mean haptoglobin concentration was between 1 and 25 μg/ml for all groups before infection. By contrast infection caused haptoglobin concentrations to rise to between approximately 500 to 2500 μg/ml at Day 5 post infection, which was a significant (P < 0.05) increase for all infected groups with the exception of the control group in study C, where only a trend was observed (P = 0.112).

**Table 2:** Serum haptoglobin concentrations (μg/ml) in mice before and after *Salmonella *challenge^a^

	N^b^	Unifected	Infected
**Study A:**			

**Control**	5	5.96 ± 2.37	514.97 ± 258.32*

**FOS**	9	1.42 ± 0.49+	1796.93 ± 268.37***++

**XOS**	7	4.05 ± 2.87	1584.67 ± 346.58***+

**Study B:**			

**Control**	7	25.52 ± 12.20	1469.57 ± 455.12*

**Beta-glucan**	6	1.56 ± 0.49	1704.18 ± 368.97***

**GOS**	6	7.54 ± 5.44	966.68 ± 283.58**

**Study C:**			

**Control**	7	17.03 ± 6.39	1384.38 ± 515.84

**Inulin**	7	9.64 ± 7.38	2369.71 ± 862.14**

**Apple pectin**	5	3.55 ± 2.83	1993.22 ± 673.85***

**Polydextrose**	5	14.82 ± 10.47	1477.68 ± 512.44*

^a^Values represent means ± SEM. ^b^Numbers of mice where serum haptoglobin was measured in uninfected and infected mice. *Significantly different from the corresponding concentration measured in uninfected mice. *P < 0.05; **P < 0.01; ***P < 0.001. +Significantly different from the concentration measured in infected mice fed the control diet. +P < 0.05; ++P < 0.01.

When comparing infected groups fed putative prebiotics with infected control groups, it was seen that for mice fed FOS and XOS, serum haptoglobin concentrations were significantly higher, P < 0.01 and P < 0.05 respectively, when compared to the control group. In the other parts of the study, it was also seen that prebiotic groups generally did not cause a lower and in most cases caused a higher haptoglobin concentration after infection compared to the control group, with the notable exception of GOS where the trend was a lower level.

### Cellular Composition of the Spleen of mice from Study C

**Figure 2:**
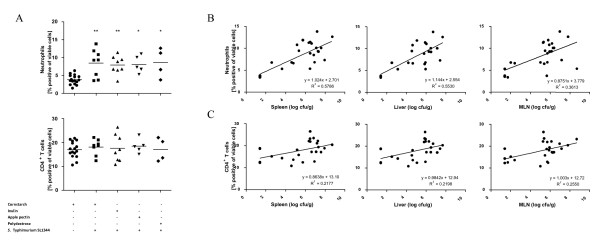
**Prevalence and linear correlations of immune cells in spleen after Salmonella challenge**. A: The percentages of neutrophils and CD4^+ ^T cells within the spleen of infected versus non-infected mice. * P < 0.05; **P < 0.01. Linear correlations between numbers of cultivated *Salmonella *from spleen, liver and mesenteric lymph nodes and prevalence of B: neutrophils and C: CD4^+ ^T cells.

To further explore the action of the immune system on *Salmonella *infection in Study C, the composition of immune cells (CD4^+ ^and CD8^+ ^T cells, NK and NKT cells, B cells, dendritic cells and neutrophils) within the spleen of non-infected as well as infected mice was analysed by flow cytometry. No significant effects of the different prebiotic feeds were demonstrated, however, a significant increase in the percentage of neutrophils (P < 0.01) within the spleen of infected mice was found, compared to non-infected controls (Figure 2A). This increase positively correlated with the numbers of *S*. Typhimurium cultivated five days post challenge from liver (P < 0.001), spleen (P < 0.001) and mesenteric lymph nodes (P < 0.01) (Figure 2B), but not from ileum (data not shown). Furthermore, a positive correlation between the percentages of CD4^+ ^T cells within the spleen of infected mice and the numbers of *S*. Typhimurium cultivated from liver (P < 0.05), spleen (P < 0.05) and mesenteric lymph nodes (P < 0.05) five days post challenge was established (Figure 2C), although the increase in CD4^+ ^T cells in infected mice was not significant.

### In vitro fermentation study

By *in vitro *fermentation using monocultures of *S*. Typhimurium, this strain was seen to utilise FOS (P < 0.01), beta-glucan (P < 0.05) and GOS (P < 0.001), but not XOS, Inulin, apple pectin or polydextrose. In accordance with these results, a lowering of the culture pH was seen after fermentation with FOS (P < 0.01), beta-glucan (P < 0.001), and GOS (P < 0.001). A significant decrease in the pH was also recorded in the culture with polydextrose (P < 0.001) even though this carbohydrate was not found to support growth of the *Salmonella *strain (data not shown).

## Discussion

In the present study we report for the first time that changes in the carbohydrate composition of the diet impair the resistance of BALB/c mice to severe *S*. Typhimurium SL1344 challenge. Mice fed with a diet containing 10% FOS or XOS had significantly higher numbers of *S*. Typhimurium in liver (P = 0.006 and P = 0.023, respectively), spleen (P = 0.010 and P = 0.025, respectively) and mesenteric lymph nodes (P = 0.009 and P = 0.017, respectively) when compared to mice fed with the control diet. Additionally, a similar trend was observed for the mice fed with apple pectin, which also had elevated numbers of *Salmonella *in liver (P = 0.154) and spleen (P = 0.198).

The haptoglobin concentrations seen in the infected mice quite closely correlated with the degree of translocation of *Salmonella*, scored as the numbers of CFU of *Salmonella *in liver, spleen and mesenteric lymph nodes in the dietary groups of each of the three experiments. Thus in Study A, the significantly increased number of *Salmonella *in the organs of the FOS and XOS groups compared to the group fed the control diet (Figure 1) correlated with haptoglobin concentrations that were significantly increased in the same groups compared to the control group (Table 2). In Study B and C, no statistically significant differences after infection were detected in either haptoglobin concentration or organ counts between the dietary groups and the control group of each experiments. Still, there was a trend for correlation between high haptoglobin concentrations and high organ counts, as seen for example for the apple pectin group of Study C (in which the haptoglobin level was most significantly increased compared to the level observed before infection) while low haptoglobin levels correlated with low organ counts as observed for the GOS group in study B.

To further explore the mechanism behind the increase in haptoglobin concentration observed post challenge with *Salmonella *in study A and B, in study C we included flow cytometric analysis of the cellular composition of the spleen. Of all the cell subsets analysed, only the proportions of neutrophils were significantly increased upon infection. We also found a positive correlation between the number of neutrophils in the spleen and the CFU of *Salmonella *in the organs of the infected mice, but not the CFU of *Salmonella *in the ileum, indicating that the neutrophil number and thus the haptoglobin concentration reflects an immune response towards the bacteria translocated to the organs rather than the *Salmonella *present in the gastrointestinal tract. This is in accordance with earlier findings demonstrating that neutrophils are important for host survival during the primary response to *Salmonella *infection, primarily due to control of bacterial replication [32]. Other investigators have reported changes in other cell subsets in the spleen post infection, e.g. a decrease in T, NK and NKT cells [33], but although there was a positive correlation between organ CFU and T cell numbers, we did not find other significant changes in the cell numbers of the different cell populations analysed.

Studies reporting adverse effects of FOS and inulin on *S*. Enteritidis infections in rats have been published [28-31]. In these studies it is hypothesised that the increased translocation of *S*. Enteritidis, measured as increased urinary excretion of nitrates and nitrites, is caused by fermentation of the prebiotics producing high concentrations of lactic acid and short chain fatty acids. This was found to impair the mucosal barrier, measured as faecal mucin excretion [28-31]. However, the studies were all based on low calcium diets (0.80-1.20 g Ca/kg) and the adverse effect could be reversed by oral administration of calcium [31]. Acidification of the gut content has been shown to be counteracted by dietary calcium, suggesting that the increased translocation could be connected to low pH [34,35]. However, the diets used in our study contained the amount of calcium recommended for rodents (5 g/kg) [36], and our results thus contradict that the observed increased translocation occurs only when the diet is low in calcium. Additionally, our results contradict that acidification *per se *should mediate the increased translocation, since no drop in cecal pH was observed in animals fed with FOS or XOS in the present study (Table 1).

The major effects of prebiotic fermentation are typically seen in the large intestine, however according to the refined definition of prebiotics [7], as well to the results presented here, the effects are not restricted to the colon. *Salmonella *translocates primarily through M cells located in the ileal Peyer's patches [37], and an increased concentration would be likely to result in an increased number of phagocytosed *S*. Typhimurium. However, even though the trends in our data indicated that a high ileal content of the pathogen was accompanied by a high amount of *Salmonella *in internal organs (Figure 1), it should be noted that consumption FOS and XOS, leading to significantly increased amounts of *Salmonella *in liver and spleen was *not *accompanied by significantly increased ileal counts of the pathogen (P > 0.20), and that apple pectin, which significantly increased ileal Salmonella counts did not lead to significantly increased numbers of this pathogen in the internal organs (P = 0.154 and P = 0.198, respectively).

With the notable exception of GOS, our data suggest that small-molecule prebiotics increase *Salmonella *translocation more than larger molecules (Figure 1). Ten Bruggencate *et al*. [31] studied the effect of FOS and inulin on *S*. Enteritidis infection in rats and reported an increase in *S*. Enteritidis translocation in rats fed a low calcium diet with FOS as well as with inulin. However, in the present study, no increased translocation of *S*. Typhimurium was observed in mice fed inulin (Figure 1C). We speculate that the effect of prebiotics on bacterial translocation may be different in rats and mice, and may also depend on the *Salmonella *serovar used, and on other dietary or environmental factors than calcium.

A recent study demonstrated that oral administration of a mixture of GOS can reduce numbers of *S*. Typhimurium SL1344 in the liver and spleen of BALB/c mice when given just prior to infection [27]. This is in contradiction to the results reported in the present paper, which show no protective effect of GOS against *Salmonella *(Figure 1). The differences may be explained by the fact that oral delivery of GOS (2500 mg/kg) was given to mice just 30 minutes prior to *Salmonella *challenge [27], as opposed to the approach chosen in the present study, which was designed to mimic how continuous ingestion of non-digestible carbohydrates (e.g. as part of a regular diet) affects susceptibility to infection.

Our findings of increased caecum weight (Table 1) in mice fed FOS, XOS or polydextrose indicate increased fermentation in caecum. However, the increase was only accompanied by a decline in caecal pH in the group fed polydextrose. In accordance with our findings, polydextrose has been reported to increase the weight of caecal dry matter, to decrease caecal pH and to change the composition of the caecal microbial community in rats [38]. Similar changes have been reported for FOS and XOS in rats with increased numbers of caecal bifidobacteria [11].

Our *in vitro *fermentation experiment showed that *S*. Typhimurium SL1344 is capable of fermenting FOS, beta-glucan, GOS and glucose with a corresponding decline in pH. Polydextrose was not found to support growth of the *Salmonella *strain, but a significant reduction in pH was recorded, indicating metabolic activity. In accordance with our observation, Ten Bruggencate *et al*. 2003 [29] stated that *Salmonella *can use FOS as a substrate for growth. Additionally, Fooks & Gibson [18] reported growth of *S*. Enteritidis on inulin, FOS and XOS, however generally with a lower specific growth rate than selected probiotic strains. In co-culture with probiotics growth of the *Salmonella *strains was significantly reduced by FOS and XOS.

The results obtained from the *in vitro *studies did not explain our *in vivo *observations. While e.g. apple pectin was not fermented by *Salmonella in vitro*, highly increased levels of ileal *S*. Typhimurium was observed in animals fed with this carbohydrate (Figure 1C). This may reflect the growth of *Salmonella *on by-products from fermentation of apple pectin or XOS by other gut bacteria. Additionally, *in vivo*, *Salmonella *competes for nutrients with the resident microbiota, of which some bacteria may be more efficient in fermenting the various carbohydrate sources than what we see for *Salmonella in vitro*. Factors such as the chain length, branching, and the type of bond linking the monomers, in view of specific enzymes required for fermentation, are likely to contribute to the *in vivo *competition. Our results thus further highlight that laboratory monocultures are not adequate for prediction of bacterial growth (or absence of growth) in the complex intestinal ecosystem.

## Conclusion

Based on the results presented within this study we conclude that changes in the carbohydrate composition of diets fed to mice alter the resistance to *S*. Typhimurium infections. This raises important doubts about the potential use of certain prebiotics for prevention of *Salmonella *infections. However, it should be kept in mind that our observations do not contradict the proposed beneficial effects of prebiotics in prevention of life-style related diseases such as colon cancer, inflammatory bowel disease and cardiovascular disease, which are likely to be affected by completely different mechanisms than those important for protection against pathogens.

## Methods

### Animals and housing

4 week-old conventional male BALB/c mice were purchased from Taconic Europe (Lille Skensved, Denmark) and housed individually in standard cages in an environmentally controlled facility with a 12-h light/dark cycle. During the study the temperature was kept at 22 ± 1°C, relative humidity at 55 ± 5% and air was changed 8-10 times per hour. Animal experiments were carried out under the supervision of the Danish National Agency for Protection of Experimental Animals.

### *Salmonella *strain

A *gfp*+ tagged *S*. Typhimurium SL1344 strain resistant to nalidixic acid and chloramphenicol was constructed and used throughout this study in order to facilitate enumeration and verification of *Salmonella *in un-sterile samples. To construct this strain, a spontaneous nalidixic acid resistant mutant of *S*. Typhimurium SL1344 (designated JB371) was initially selected. Next, the genetic element P*rpsM'*-*gfp*^+^-*cat *of strain JH3016 [33] was introduced into the chromosomally located *putPA *region of strain JB371 by P22 transduction using a P22 lysate of strain JH3016 (kindly provided by Isabelle Hautefort, Norwich, UK). The resulting *gfp*+ tagged *S*. Typhimurium SL1344 strain resistant to nalidixic acid and chloramphenicol was designated JB400 (designated *S*. Typhimurium throughout the paper).

### Dietary Carbohydrates

Inulin, DP 2-60 (Orafti ST-Gel, Beneo-Orafti, Tienen, Belgium) and FOS, DP 2-8 (Orafti P95, Beneo-Orafti, Tienen, Belgium) were purchased from Alsiano, Birkeroed, Denmark. XOS, DP 2-6, GOS, DP 2-6, and polydextrose with an average DP of 12 were kindly provided by Danisco Health & Nutrition, Kantvik, Finland. Apple pectin was purchased from Obipektin AG, Bischofszell, Switzerland and beta-glucan (Glucagel™ 75) was purchased from GraceLinc Limited, Christchurch, New Zealand.

### Challenge protocol

*S*. Typhimurium SL1344 was grown in closed 50 ml tubes at 37°C, 200 rpm overnight in 20 ml LB broth supplemented with 10 μg/ml chloramphenicol. Overnight cultures were diluted to 10^8 ^CFU/ml in saline and animals were orally infected with 0.1 ml (10^7 ^CFU) by gastric gavage. The number of CFU in the inoculum was determined by plating on LB-agar plates supplemented with 10 μg/ml chloramphenicol. The inoculum size was chosen based on a series of pilot-experiments determining the dose-response of this particular strain in the animal model.

### Diets and experimental design

For an acclimatisation period of 1-2 weeks prior to commencement of the feeding experiments the mice were fed a standard mouse diet produced in house as previously described [39] based on the rodent diet AIN-93 [36] containing cornstarch as the major carbohydrate source. Subsequently, the mice were randomised to 8 dietary groups with 8 mice per group (10 in the FOS group). The experimental diets based on AIN-93 were supplemented with 10% of either of the following carbohydrates: fructo-oligosaccharide (FOS), xylo-oligosaccharide (XOS), beta-glucan, galacto-oligosaccharide (GOS), inulin, apple pectin or polydextrose in place of an equal amount (w/w) of cornstarch. Three independent studies were carried out with a cornstarch-based diet as control: Study A: Control, FOS and XOS; study B: Control, beta-glucan and GOS; study C: Control, inulin, apple pectin and polydextrose). Diets and water acidified with citric acid to pH 3.0 to prevent growth of microorganisms were provided ad libitum.

Mice were fed the respective diets for three weeks prior to *Salmonella *challenge and body weight was recorded weekly. Following the three weeks all mice were challenged with 10^7 ^CFU *S*. Typhimurium SL1344 and scheduled for euthanisation on Day 5 after challenge. The mice were kept on their respective diets and observed twice a day. If symptoms of severe disease (ruffled fur, changed behaviour) developed, the mice were euthanised immediately due to ethical considerations.

On the day of euthanisation the mice were dissected and *S*. Typhimurium SL1344 was cultivated from the liver, spleen, mesenteric lymph nodes and content of the distal part of ileum. The weight (with content) and pH of caecum were recorded for each mouse. In the study with FOS and XOS the caecal content was diluted 3× in sterile water before pH was measured.

### *Salmonella *cultivated from organs, content of distal ileum and faecal samples

Liver, spleen, mesenteric lymph nodes and content of the distal part of ileum were 10-fold diluted in saline and homogenised. Serial dilutions of the homogenates were plated on LB-agar plates containing 10 μg/ml chloramphenicol. The plates were incubated aerobically at 37°C overnight. Faecal samples (wet weight) were collected from mice on Days 1, 3 and 5 after *Salmonella *challenge and cultivated as described for the organ samples.

### Measurement of serum haptoglobin concentrations

Blood samples were taken from all mice one week prior to *Salmonella *challenge and on the day of euthanisation for analysis of the acute phase protein haptoglobin. Haptoglobin has been described as a highly reactive acute phase protein in mice [40] whereas for example C-reactive protein is not a prominent acute phase protein in the mouse [41]. The samples were stored overnight at 5°C and centrifuged at 3000 rpm for 20 minutes for isolation of serum. Serum samples were stored at -20°C. Buffers used for the haptoglobin determination were PBS/T (0.05% (v/v) Tween 20 in PBS) and PBS/T/BSA (0.05% (v/v) Tween 20 in PBS, 1% BSA (Sigma-Aldrich A2153)). All chemicals were from Sigma-Aldrich, all incubation volumes were 100 μl/well and incubations were at room temperature, unless otherwise indicated. ELISA plates (NUNC MaxiSorp) were coated with rabbit anti human haptoglobin (DAKO A030) diluted 1:10000 in 0.1 M sodium hydrogencarbonate pH 9.6 and stored overnight at 5°C. Plates were washed four times in PBS/T, blocked with PBS/T/BSA (200 μl/well) and incubated for 30 minutes. Plates were then washed as before and loaded with a mouse haptoglobin standard (RS-90HPT, Gentaur Molecular Products, Belgium) diluted 1:2000 in PBS/T/BSA and applied in six 2-fold dilutions (each dilutions applied in two wells). Serum samples were also determined in duplicate, and diluted in PBS/T/BSA. After incubation for one hour, plates were washed as above and then incubated with biotinylated A030 diluted in PBS/T/BSA for one hour followed by washing as before. A030 was biotinylated by incubation at pH 8.2 with biotin-N-hydroxysuccinimide (approximately 100 μg/mg immunoglobulin), followed by dialysis against PBS. Finally, plates were incubated with peroxidase-conjugated streptavidin (DAKO P397) diluted 1:5000 in PBS/T/BSA for one hour, washed as before and stained with tetramethyl benzidine/peroxide substrate (TMB PLUS from Kem-En-Tec, Denmark). The reaction was stopped by adding 100 μl 0.5 M H_2_SO_4 _to each well and the optical density at 450 nm corrected for background optical density at 650 nm was recorded using a dedicated ELISA reader (Thermo Multiskan Ex spectrophotometer, Thermo Scientific, Waltham, MA, USA). All samples including standards were determined in duplicate. Sample values were calculated from the curve fitted to the readings of the standard (using Ascent software v. 2.6, Thermo Scientific). The detection limit of the assay was 0. 5 μg/ml.

### Immunocytostaining and Flow Cytometry

Single-cell suspensions were prepared from spleens and transferred to round-bottomed 96-well polystyrene plates (NUNC, Roskilde, Denmark) with 3 × 10^5 ^cells/well. Fcγ III/II (3 μg/ml, 50 μg/ml; BD Biosciences) was added for 10 minutes to block non-specific binding of antibodies. An additional 50 μl/well PBS-Az containing fluorochrome-conjugated antibodies at various concentrations was added and the cells were incubated for 45 minutes. The cells were then washed and resuspended in 200 μl/well PBS-Az containing 2% formaldehyde for flow cytometric analyses. All stainings were carried out at or below 4°C. The antibodies used in this study were APC-conjugated anti-mouse CD4, clone RM4-5 (rat IgG2a,κ); PE-conjugated anti-mouse CD3e, clone 145-2C11 (Armenian hamster IgG); APC-conjugated anti-mouse CD8a (Ly2), clone 53-6.7 (rat IgG2a, κ); APC-conjugated anti-mouse CD49b, clone DX5 (rat IgM, κ); PE-conjugated anti-mouse CD19, clone 1D3 (rat IgG2a, κ); APC-conjugated anti-mouse CD11c, clone N418 (Armenian hamster IgG); APC-conjugated anti-mouse Ly-6G (Gr-1), clone RB6-8C5 (rat IgG2b, κ) and isotype controls for rat IgG2a, κ; rat IgG2b, κ; Armenian hamster IgG1, clone eBio299Arm; rat IgM, κ, all purchased from eBioscience. Stained cells were analysed on a BD FACSArray flow cytometer (BD Biosciences) and data was analysed using FCS Express 3.0 software (De Novo Software, CA).

### *In vitro *fermentation of non-digestible dietary carbohydrates

The fermentation study was performed using a basal medium containing: peptone water (2 g/L, Oxoid), yeast extract (2 g/L, Oxoid), NaCl (0.1 g/L, Merck), K_2_HPO_4 _(0.04 g/L, Merck), KH_2_PO_4 _(0.04 g/L, Merck), MgSO_4_·7H_2_O (0.01 g/L, Merck), CaCl_2_·6H_2_O (0.01 g/L, Sigma-Aldrich), NaHCO_3 _(2 g/L, Merck), haemin (0.005 g/L, Sigma-Aldrich), L-cystein HCL (0.5 g/L, Sigma-Aldrich), bile salts (0.5 g/L, Oxoid), Tween 80 (2 ml/L, Merck), vitamin K_1 _(10 μl/L, Sigma-Aldrich), resazurin (0.001 g/L, Sigma-Aldrich) and 1% (wt/vol) test carbohydrate (inulin, FOS, XOS, GOS, beta-glucan, apple pectin, polydextrose and glucose) [42].

Stock solutions of peptone water, NaCl, K_2_HPO_4_, KH_2_PO_4_, CaCl_2_·6H_2_O, MgSO_4_·7H_2_O and NaHCO_3 _were prepared and autoclaved (121°C, 15 min.). Appropriate volumes of the stock solutions were mixed, autoclaved and supplemented with sterile filtered (0.2 μm) solutions of bile salts, L-cystein HCL, resazurin and yeast extract. Furthermore, haemin, Tween 80 and vitamin K_1 _were added. Stock solutions of the test carbohydrates were prepared by autoclaving (XOS, beta-glucan and apple pectin) or by sterile filtration (inulin, FOS, GOS, polydextrose and glucose).

An overnight culture of *S*. Typhimurium SL1344 (cultivated in 20 ml LB broth supplemented with 100 μg/ml nalidixic acid) was centrifuged at 1500 g for 30 minutes at 5°C and re-suspended in basal medium. The culture was inoculated in basal medium supplemented with test carbohydrates to an initial OD_600 _of 0.01. The fermentation study was performed under anaerobic conditions at 37°C, 200 rpm for 24 hours with recording of the initial and 24 h OD_600 _and pH values. A positive control (glucose) and a blank control with no additional carbon source added were included in the study. The sterility of the basal medium and carbohydrates was tested by incubation without bacterial inoculation. pH was measured before and after fermentation. Growth on a given carbohydrate was defined as significant difference from the OD600 measured in the blank sample after fermentation. All fermentations were performed in triplicate.

### Statistical analysis

All parameters were analysed using a one-way analysis of variance (ANOVA). Where ANOVA indicated a significant difference Student's t-test was used to compare dietary groups with control. All statistical analyses were carried out using SAS JMP 6.0.2. *P *values of < 0.05 were considered statistically significant.

## Authors' contributions

All authors were part of a project group, which continuously followed and discussed the progress of the experiments. AP designed and carried out the animal studies, performed the statistical analysis and drafted the manuscript. TRL and HF conceived of the study and participated in its design and coordination as well as in the preparation of the manuscript. ALP carried out the *in vitro *fermentation study, PMHH carried out the haptoglobin determination, JBA performed the fluorescent tagging of the *Salmonella *strain, RBS performed the immunocytostaining and flow cytometry, and MP contributed to feed design and statistical analysis. SJL and AO contributed significantly to the interpretation of data and the preparation of the manuscript. All authors read and approved the final manuscript.
